# Maxillary Antrolith: A Rare Cause of the Recurrent Sinusitis

**DOI:** 10.1155/2013/527152

**Published:** 2013-02-07

**Authors:** Vijendra Shenoy, Vinod Maller, Vijetha Maller

**Affiliations:** ^1^Department of ENT and Head, and Neck Surgery, Kasturba Medical College, Manipal University, Mangalore, Karnataka 575001, India; ^2^Department of Otolaryngology, Kasturba Medical College Hospital, Manipal University Attavar, Mangalore, Karnataka 575001, India; ^3^Department of Radiology, Kasturba Medical College, Manipal University, Mangalore, Karnataka 575001, India

## Abstract

*Introduction*. An antrolith is a calcified mass within the maxillary sinus. The origin of the nidus of calcification may be extrinsic (foreign body in sinus) or intrinsic (stagnant mucus and fungal ball). Most antroliths are small and asymptomatic. Larger ones may present as sinusitis with symptoms like pain and discharge. *Case Report*. We report a case of a 47-year-old lady who presented with heaviness on the left side of the face and loosening of the left 2nd molar tooth since two months. CT scan of the osteomeatal complex and paranasal sinuses showed an opacification of bilateral maxillary sinus and an amorphous area of bone density in the left maxillary sinus. Because of the size of the mass, benign neoplasms were considered in the differential diagnosis. During an endoscopic sinus surgery, it was found to be an antrolith, which was successfully managed by antrostomy and Caldwell-Luc Surgery. *Discussion*. Antrolith is a rare condition. Rhinoliths are known to invade into the maxillary antrum, but a localised lesion in the antrum is very unusual. A case of an isolated antrolith is presented for its rarity and for differential diagnosis of localised antral disease. *Conclusion*. Antrolith should be considered as differential diagnosis of unilateral radio-opaque paranasal sinus lesions.

## 1. Introduction

Antrolith is a calcified mass that occurs in the maxillary sinus. Stones arising in the antral cavities are uncommon, and their development is similar to that of a sialolith. They may form around a nidus or a concentrated mucus, which continues to grow because of the precipitation of calcium salts in concentric layers [1, 2]. Smaller antroliths are usually asymptomatic and may be discovered incidentally on routine radiography of the region [3].

## 2. Case Report

A 47-year-old lady presented to us with heaviness on the left side of the face since two months and loosening of the left 2nd molar tooth of recent onset. She had also noticed a foul smelling purulent nasal discharge from the left nostril since the past week. Her past history records revealed that she had undergone Caldwell-Luc surgery on the left side way back in the year 1984 for the removal of unilateral extensive polypoid disease. The family and personal history were not significant.

**Figure 1:**
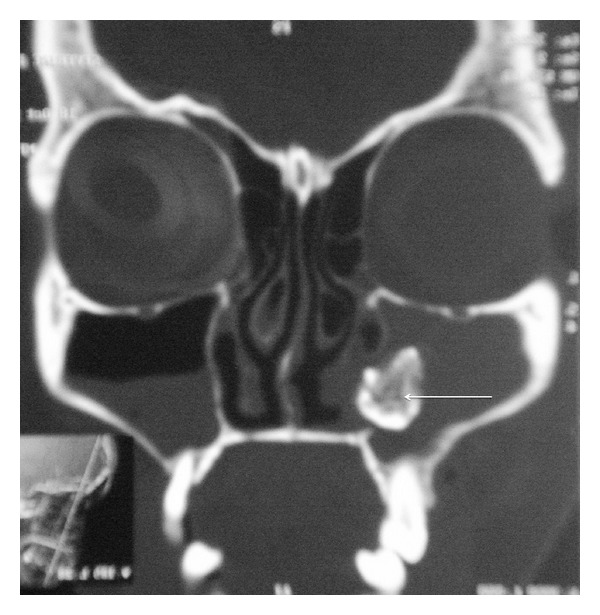
Coronal CT OMC showing antrolith.

**Figure 2:**
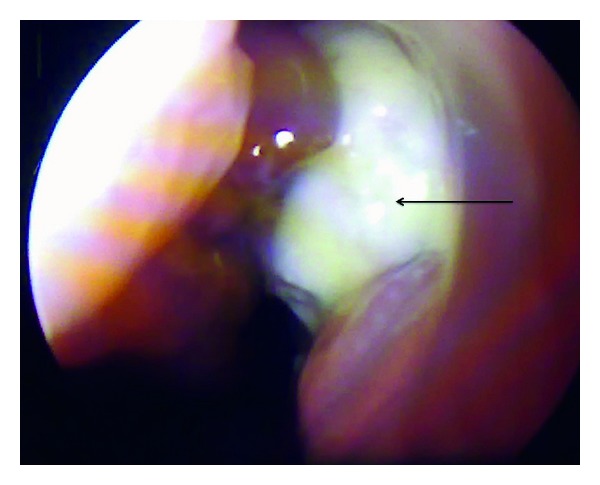
Diagnostic nasal endoscopy showing thick purulent discharge in the middle meatus.

**Figure 3:**
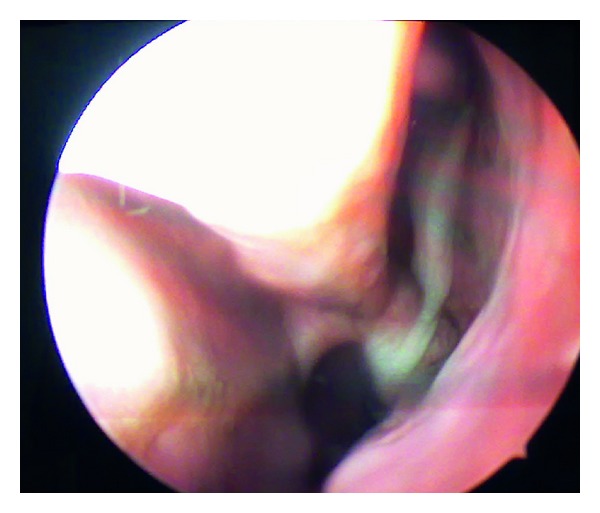
Diagnostic nasal endoscopy showing thick pus trickling down into the nasopharynx.

 Her general physical examination appeared normal without any obvious deformities or abnormalities. Oral cavity examination revealed the presence of a mobile upper 2nd molar tooth on the left side along with the presence of purulent discharge beside the teeth suggestive of an oroantral fistula. Posterior pharyngeal wall showed the presence of a thick postnasal drip. Her nasal cavity and paranasal sinus examination were essentially normal but for a mid-level deviation in the septum and tenderness elicited on the skin overlying the left maxillary sinus. All of her other systems seemed to be normal clinically. So with a diagnosis of recurrent maxillary sinusitis and an oroantral fistula in mind, we proceeded with plain computed tomography scan of the osteomeatal complex and the paranasal sinus, which showed opacification of bilateral maxillary sinus and amorphous area of bone density in the left maxillary sinus suggestive of osteoma (Figure 1). Keeping the clinical picture in mind, we also thought of a primary malignancy of the maxillary sinus. Prior to any operative intervention, a thorough diagnostic nasal endoscopy was performed which revealed thick purulent discharge in the middle meatus (Figures 2 and 3). All the hematological and biochemical investigations were within normal range.

**Figure 4:**
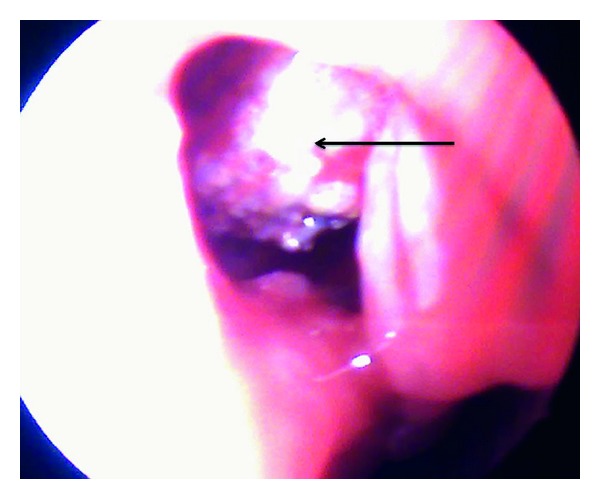
A wide middle meatal antrostomy revealing antrolith.

**Figure 5:**
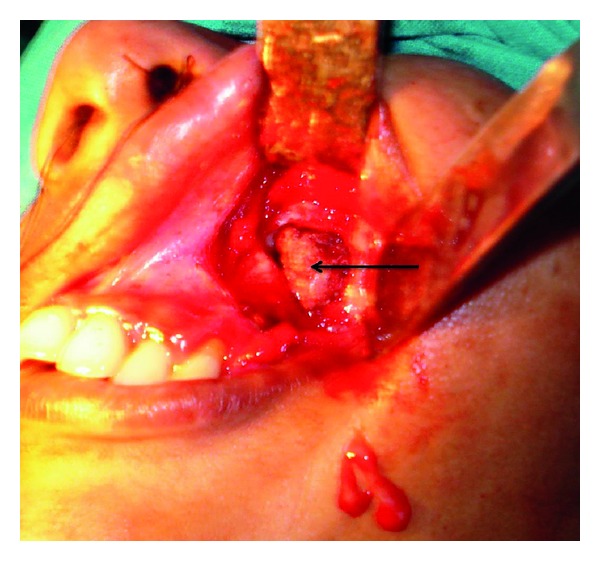
Caldwell-Luc operation revealing the antrolith.

**Figure 6:**
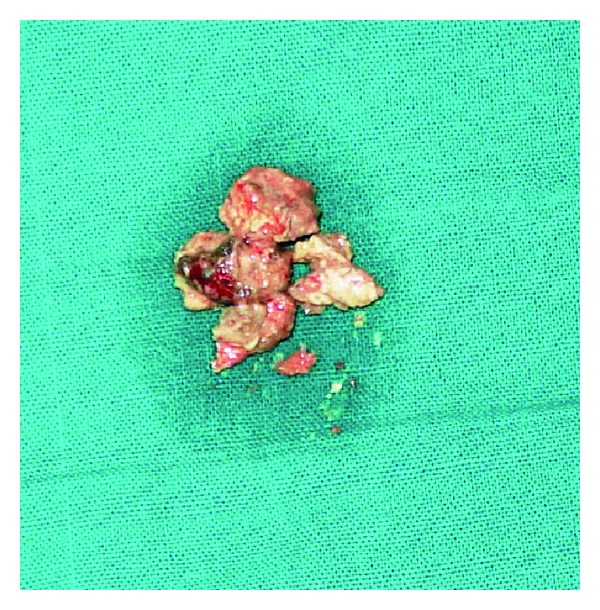
Postoperative specimen.

In order to alleviate the symptoms and reach upon a definitive diagnosis, we planned an endoscopic sinus surgery. A wide antrostomy was performed which revealed a brownish looking hard gritty mass surrounded by pus and polypoid mucosa (Figure 4). Upon probing, the mass was freely mobile with no attachment to the antral wall. Since it was difficult to remove the antrolith endoscopically via the antrostomy, a repeat Caldwell-Luc procedure was done, and calculi measuring 2 × 1 cm was removed and sent for histopathology (Figures 5 and 6). An Endoscopic sinus surgery was performed on the other side. The patient was started on intravenous cefuroxime, and the patient recovered in the postoperative period uneventfully. 

Culture and sensitivity of the pus from the left maxillary sinus revealed growth of *klebsiella* spp.resistant to ampicillin and sensitive to most of the other parenteral antibiotics. The reporting on the intraoperative specimen turned out be a diagnosis of exclusion, since the possibilities of osteoma, malignancy, rhinoscleroma, and fungal concretions were ruled out after suitable decalcification procedures and Periodic acid-Schiff staining techniques. We were thus left a freely lying calcareous mass which was irregular in shape and not attached to any wall of the maxillary sinus, and thus logically we concluded it to be a sinolith/rhinolith in the left maxillary sinus, rephrased as an antrolith. This was confirmed by the effective decalcification of the mass which left behind only the organic matter.

## 3. Discussion

Antroliths are calcified bodies within the antral cavity. The term rhinolith was first coined in 1845 to describe a partially or completely encrusted foreign body in the nose [1]. The occurrence of true antroliths is very rare, and only a total of 30 cases have been reported in the literature up until 2005 [2, 3]. The most commonly involved sinus is the maxillary sinus, followed by the frontal sinus [2]. Rhinoliths almost always occur unilaterally. Kharoubi reported an unusual case of bilateral rhinolithiasis subsequent to destruction of the posterior nasal septum [4]. 

The antral foreign body constitutes the central core of the antrolith. The central core is usually of endogenous and less commonly of exogenous origin. If the central core arises around normal or abnormal body tissues, it is of endogenous origin. These include tooth and bony fragments, blood, pus, mucus, and fungi [2]. On the other hand, if the nidus for calcification originates outside the body, then it is of exogenous origin. Exogenous niduses can be composed of different materials, such as cotton, cellulose [5], paper [6], snuff [7], dental burs [8], dental implants [9], GP points, and silver points [10]. More bizarre foreign bodies include bullets [11], pieces of glass, stones [12], wood [13], grasses, match sticks [14], sand [15], and a living leech [16]. There are reports of root canal overfilling to the maxillary sinus causing sinusitis [17]. After tooth extraction, an oroantral fistula cannot be immediately detected if the Valsalva test is not performed [18]. After healing, the oroantral fistula is small and is undetectable during the impression procedures. Zinc oxide-eugenol paste passes through the fistula in its plastic form and after curing becomes a foreign body inside the maxillary sinus. The diagnosis is only made when the patient presents the clinical symptoms of sinusitis [19]. Provided that the endonasal mucosa is intact, any tiny particles that may enter the nose during inspiration are eliminated through the secretion of mucus and ciliary action. If the mucosa is damaged, such particles may remain in the nasal cavity and grow in size through accretion of mineral salts and incrustation [20]. The pathogenesis of stone formation within a paranasal sinus is not fully understood. But, the most important predisposing factors seem to be long-standing infection, poor sinus drainage, and the presence of a foreign body in the sinus. The purulent fluid then becomes concentrated, and mineral salts, especially calcium phosphate and calcium carbonate, precipitate. As a result, complete or partial encrustation of the antral foreign body takes place [2].

 Patients with antrolith may be asymptomatic and may be incidentally discovered on routine radiological examination [21]. However, the usual clinical features in symptomatic patients are facial pain, nasal obstruction, epistaxis, purulent or blood-stained discharge, foul smelling postnasal drip, and oroantral fistula [2]. However, dacryocystitis, otorrhoea, anosmia, palatal perforation, and septal perforation have been reported in the literature [20].

Radiographically, a dense, irregular yet well-defined mass can be identified in the antrum. They can be seen on panoramic, periapical, and Waters' radiographs in addition to computed tomograms [6]. Focal antral calcification also has been seen in sinuses filled with a fungal ball of *Aspergillus fumigatus* (noninvasive mycetoma) [7]. Antroliths must be included in the differential diagnosis of radiopacities found in or near the maxillary sinus region. Other possible diagnoses can be supernumerary tooth, root fragments, osteoma, complex odontoma, mature cementoma, a periapical condensing osteitis, a buccal exostosis, a palatine torus, an impacted tooth, foreign bodies, and even neoplasms in cases of large calcified masses of the antral area [3, 8]. The management of antrolith should include surgical removal of stone by an endoscopic sinus surgery with or without Caldwell-Luc operation, along with appropriate treatment of sinus infection. 

In our case, the predisposing factor would have been bony chips left behind following the past Caldwell-Luc surgery. Thus, we suggest the thorough irrigation of sinus cavity following endoscopic sinus surgery to prevent future formation of antrolith around any endogenous nidus.

## 4. Conclusion

Although rare, antrolith should be considered as a differential diagnosis of radiopacity in the paranasal sinus lesion. An endoscopic sinus surgery combined with Caldwell-Luc operation is a reliable procedure for the removal of a large antrolith in the maxillary sinus, as it provides better exposure, ventilation, and drainage of the sinuses.
